# Supplementary material for: Interleukin-9 production by type 2 innate lymphoid cells induces Paneth cell metaplasia and small intestinal remodeling
Source: Nat Commun. 2023 Dec 2;14:7963. doi: 10.1038/s41467-023-43248-5 (PMC10693577; doi:10.1038/s41467-023-43248-5)
Supplement: Supplementary file 1 — Supplementary Information [file 41467_2023_43248_MOESM1_ESM.pdf]

SUPPLEMENTARY INFORMATION FILES

INTERLEUKIN-9 PRODUCTION BY TYPE 2 INNATE LYMPHOID CELLS INDUCES PANETH CELL METAPLASIA AND SMALL INTESTINAL REMODELING

Cheng-Yin Yuan, Aditya Rayasam, Alison Moe, Wenwen Xu, Michael Hayward, Clive Wells, Aniko Szabo, Andrew Mackenzie, Nita Salzman and William R. Drobyski

Supplemental Figure 1

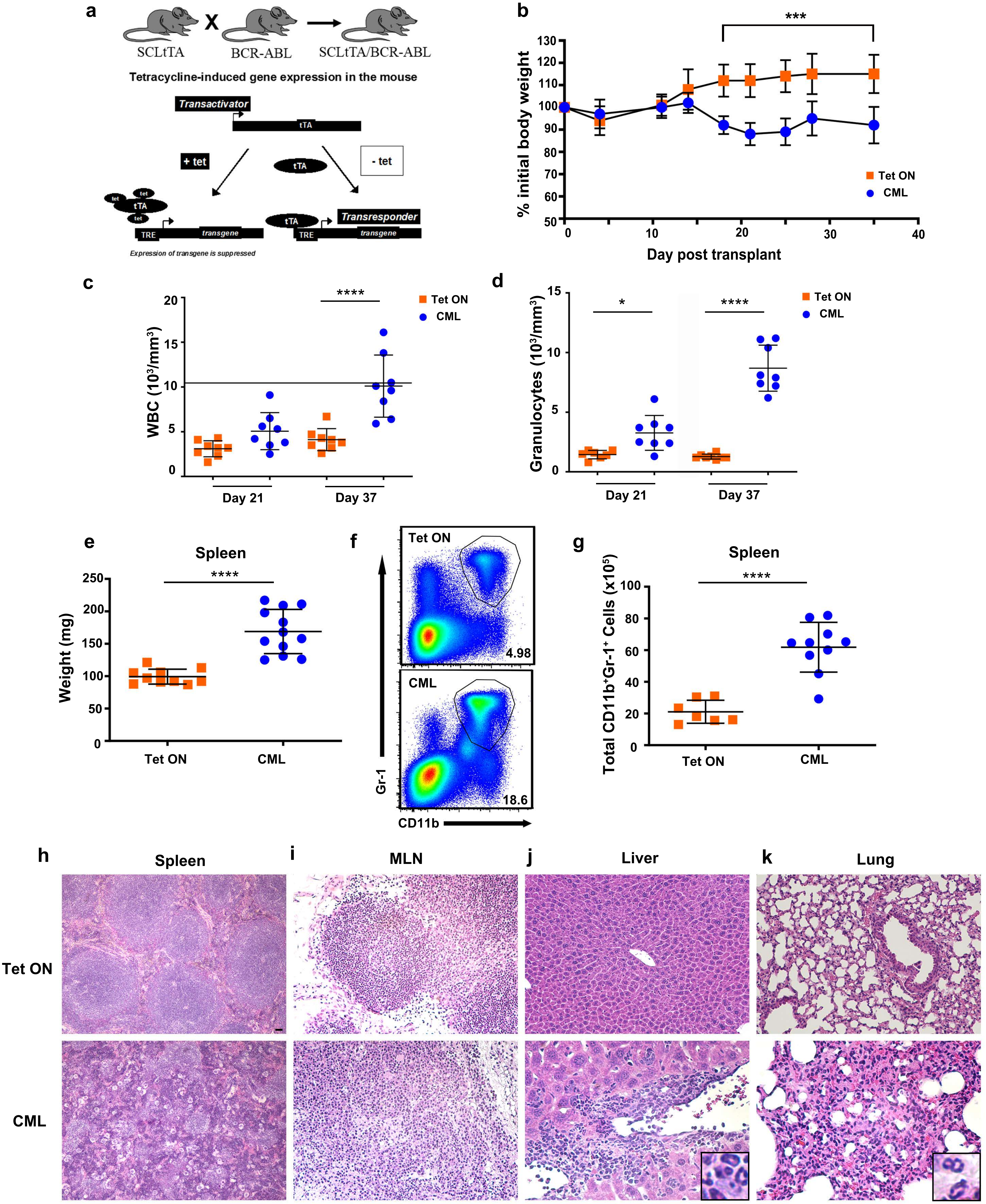

Supplemental Figure 1: **Tetracycline-off inducible murine transplantation model of CML.** (**a**) Breeding schematic and mechanism by which crossing of SCLtTa and bcr-abl mice creates mice that develop CML after withdrawal of tetracycline (Tet) from the drinking water. (**b-k**). Lethally irradiated (1100 cGy) FVB mice were transplanted with 10 x 10^6 BM cells from SCLtTa/bcr-abl animals and maintained on or off Tet in the drinking water. (**b**). Serial weight curves of mice that were maintained on or off Tet. (**c,d**). Serial total white blood cell and granulocyte counts in the peripheral blood at days 21 and 37 after transplantation. Horizontal lines in panel c denote the limits of normal for white blood cell counts. Data in panels b-d are from two experiments (n=8 mice/group). (**e**). Spleen weight 28-35 days post transplantation. Results are from three experiments (n=10-12 mice/group). (**f**). Representative dot plot depicting CD11b and Gr-1 expression on spleen cells. (**g**). Absolute number of $CD11b^+$ $Gr\text{-}1^+$ cells in the spleen 28-35 days post transplantation. Data are from three experiments (n=7-12 mice/group). (**h-k**). Representative photomicrographs of spleen, mesenteric lymph node, liver and lung from mice with and without CML 35-45 days post transplantation. Insets in panels **j** and **k** depict collections of neutrophils in liver and lung, respectively. Scale bar is 30 µM. Data are shown as mean ± SD. A mixed effects statistical model was used to compare weight curves. Pairwise statistical comparisons were performed with the Welch's t test. *p<0.05, ***p<0.001, ****p<0.0001. Panel A is reprinted from Das R, Komorowski R, Hessner MJ, Subramanian H, Huettner CS, Cua D, Drobyski WR; Blockade of interleukin-23 signaling results in targeted protection of the colon and allows for separation of graft-versus-host and graft-versus-leukemia responses; Blood, Vol 115(25): 5249-5258, 2010 with permission from Elsevier.

Supplemental Figure 2

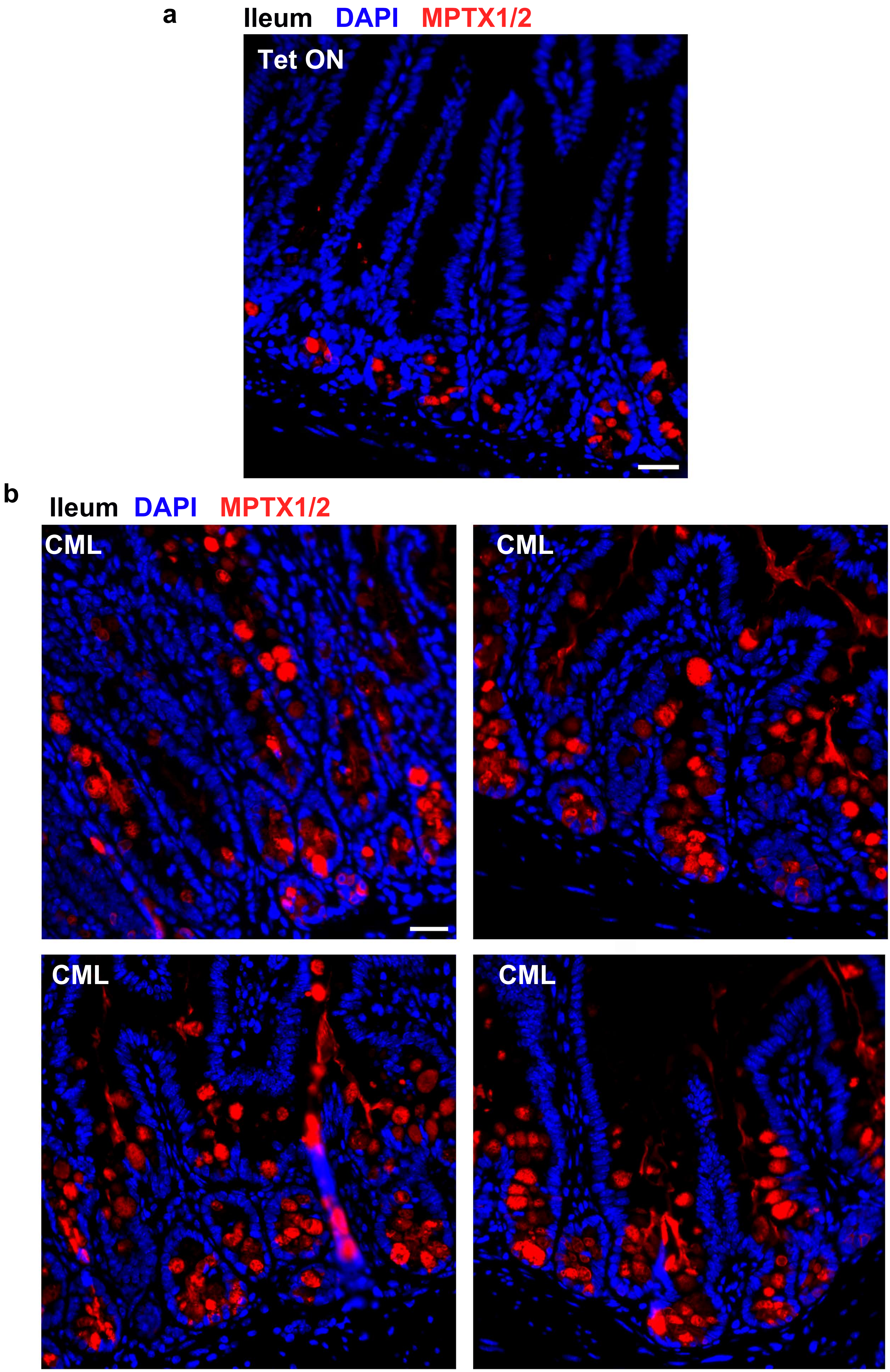

Supplemental Figure 2: **Expression of MPTX 1/2 in Paneth cells in the ileum of CML mice.** (**a,b**). Immunofluorescence staining of MPTX 1/2 in Paneth cell granules present in the ileum of lethally irradiated FVB mice transplanted with 10 x 10^6 BM cells from SCLtTa/bcr-abl mice and then maintained on Tet (panel **a**) or off Tet (CML) (panel **b**). Magnification is 20X. Scale bar is 30 µM.

Supplemental Figure 3

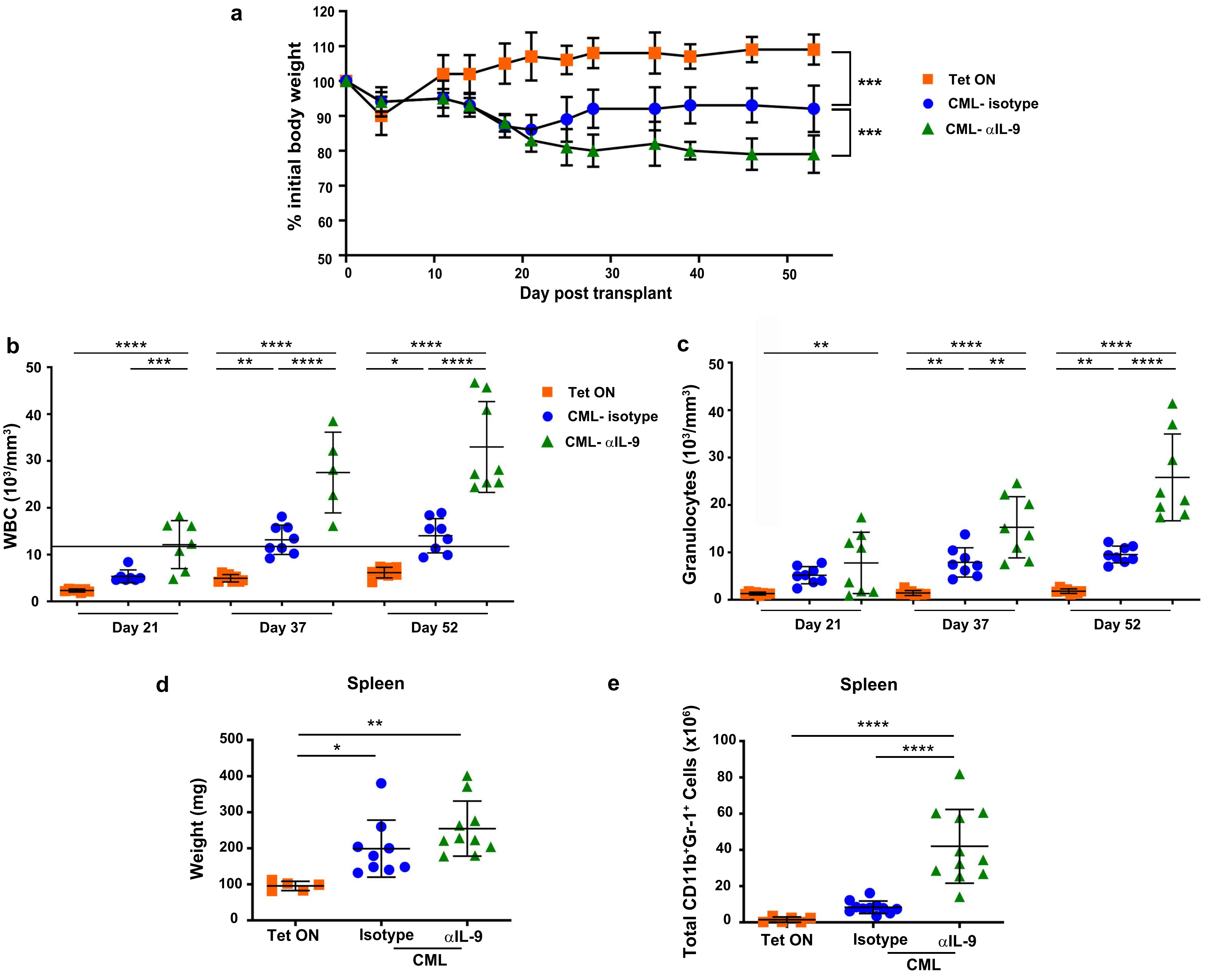

Supplemental Figure 3: **Blockade of IL-9 signaling exacerbates CML.** (**a-e**). Lethally irradiated FVB mice were transplanted with 10 x 10^6 BM cells from SCLtTa/bcr-abl mice and then maintained on Tet or taken off Tet and treated with an isotype control or anti-IL-9 antibody. (**a**). Serial weight curves are shown. (**b,c**). White blood cell (panel **b**) and granulocyte counts (panel **c**) in the peripheral blood. Data in panels A-C are from two experiments (n=8/group). (**d,e**). Overall weight and absolute number of CD11b$^+$ Gr-1$^+$ cells in the spleens of mice 30-35 days post-transplant. Results in panel **d** and **e** are from 3 experiments (n=5-10 mice/group). Data are shown as mean ± SD. A mixed effects statistical model was used to compare weight curves. Three-way statistical comparisons were performed with a one-way ANOVA with Fisher's LSD test. *p<0.05, ** p<0.01, ***p<0.001, ****p<0.0001.

Supplemental Figure 4

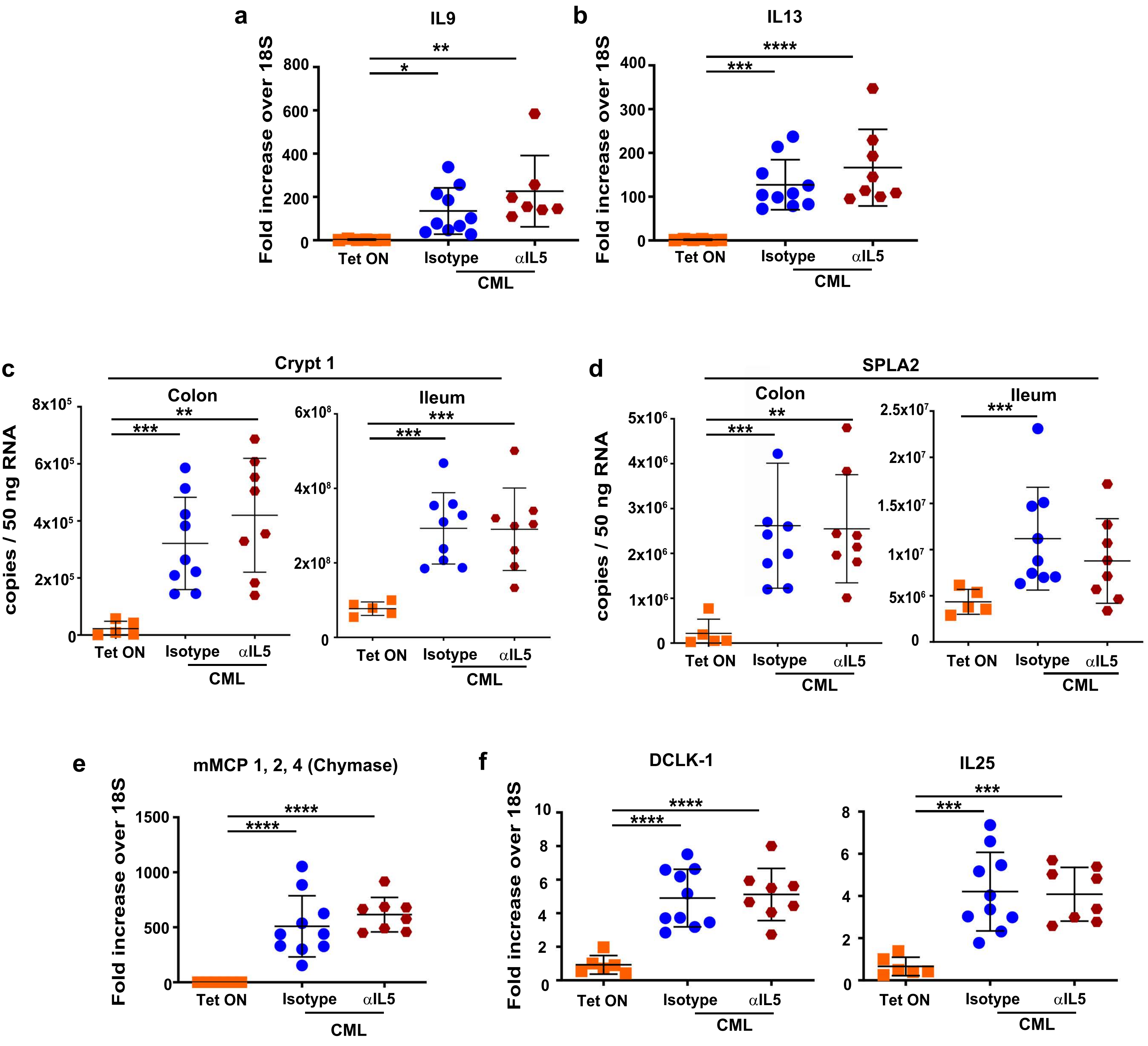

Supplemental Figure 4: **Blockade of IL-5 signaling has no effect on the expansion of specialized epithelial cell populations in the GI tract of CML mice.** (**a-f**). Lethally irradiated FVB mice were transplanted with BM from SCLtTa/bcr-abl mice. Animals were either maintained on or taken off Tet and then treated three times per week with an isotype control or anti-IL-5 antibody. (**a,b**). mRNA expression of IL-9 (panel **a**) and IL-13 gene expression (panel **b**) in the ileum. (**c,d**). mRNA expression of Crypt1 (panel **c**) and sPLA2 gene expression (panel **d**) in the colon and ileum post transplantation. (**e,f**). mRNA expression of mMCP 1,2,4 (panel **e**) and DCLK-1 and IL-25 (panel **f**) in the ileum of mice. Analysis for these endpoints was performed 23-27 days post transplantation. Data in panels **a-f** are from two experiments (n= 5-10 mice/group). Data are shown as mean ± SD. Three-way statistical comparisons were performed with a one-way ANOVA with Fisher's LSD test. *p<0.05, ** p<0.01, ***p<0.001, ****p<0.0001.

Supplemental Figure 5

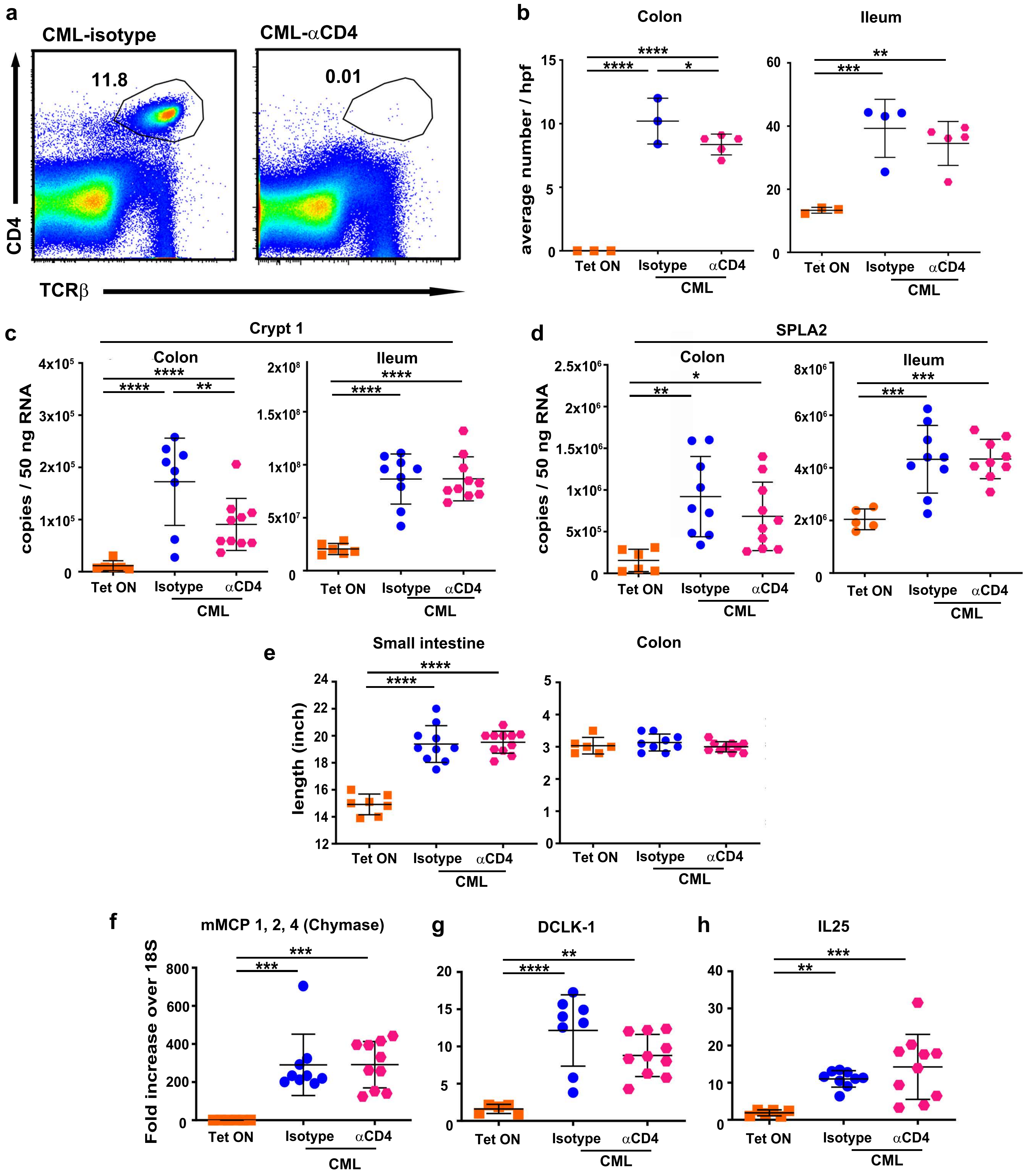

Supplemental Figure 5: **Depletion of $CD4^+$ T cells has no effect on PCM or intestinal remodeling.** (**a-h**). Lethally irradiated FVB mice were transplanted with BM from SCLtTa/bcr-abl mice. Animals were either maintained on or taken off Tet and then treated two times per week with an isotype control or anti-CD4 antibody for 28 days. (**a**). Dot plot demonstrating $CD4^+$ $TCR\beta^+$ T cells in the spleen of mice treated with an isotype control or anti-CD4 antibody. (**b**). Absolute number of Paneth cells in the colon and ileum as determined by H&E staining. Results are from one experiment (n=3-5 mice/group). (**c,d**). Crypt1 and sPLA2 gene expression in the colon and ileum. (**e**). Small intestines and colon length. (**f-h**). mRNA expression of mMCP1,2,4 (panel **f**), DLCK-1 (panel **g**), and IL-25 (panel **h**) in the ileum. Data in panels **c-h** are from two experiments (n=5-10 mice/group). Data are shown as mean ± SD. Three-way statistical comparisons were performed with a one-way ANOVA with Fisher's LSD test. *p<0.05, ** p<0.01, ***p<0.001, ****p<0.0001.

Supplemental Figure 6

a

CML- colon

DAPI E-Cadherin Cleaved Caspase-3

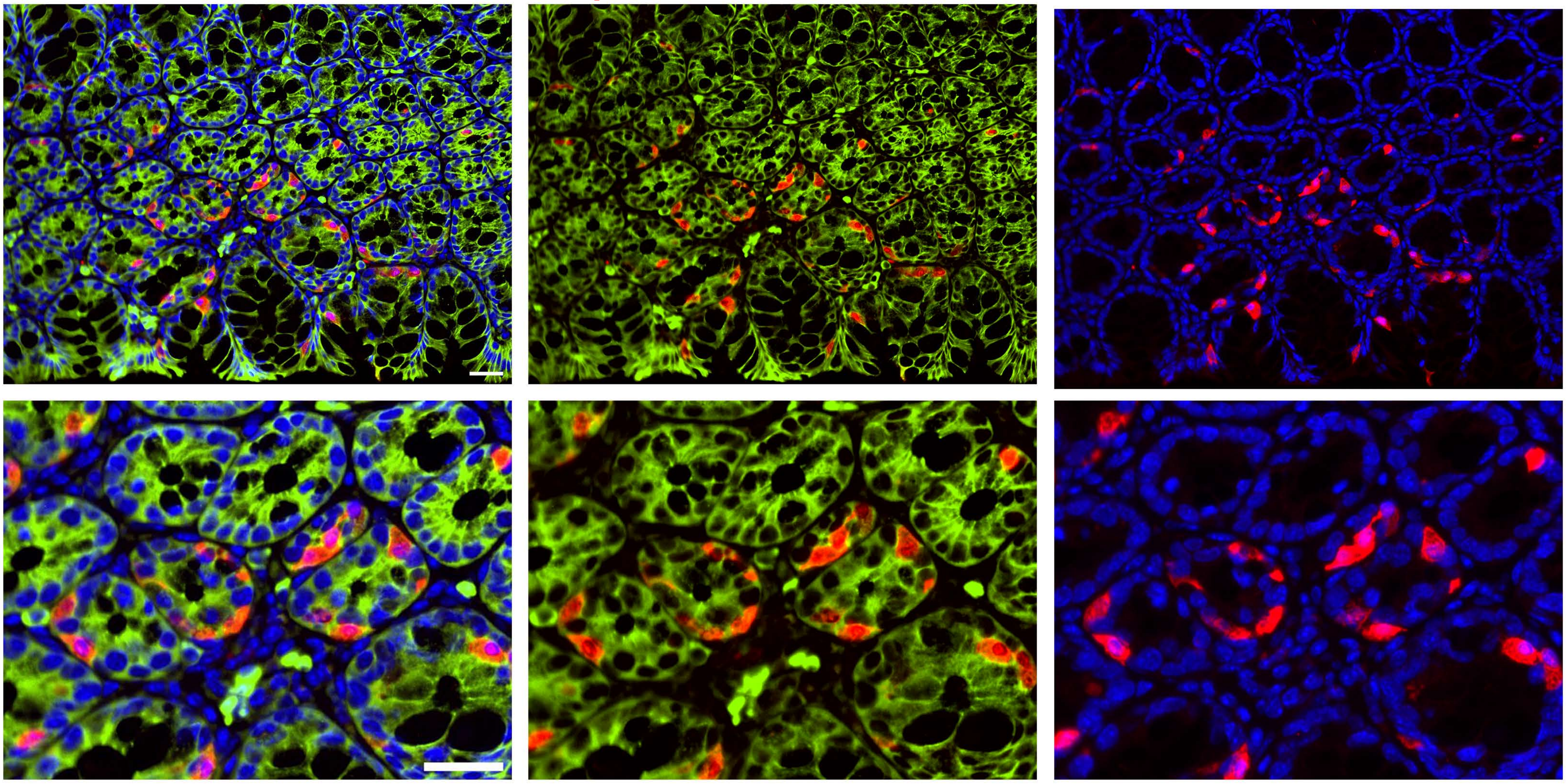

b

CML -ileum

DAPI E-Cadherin Cleaved Caspase-3

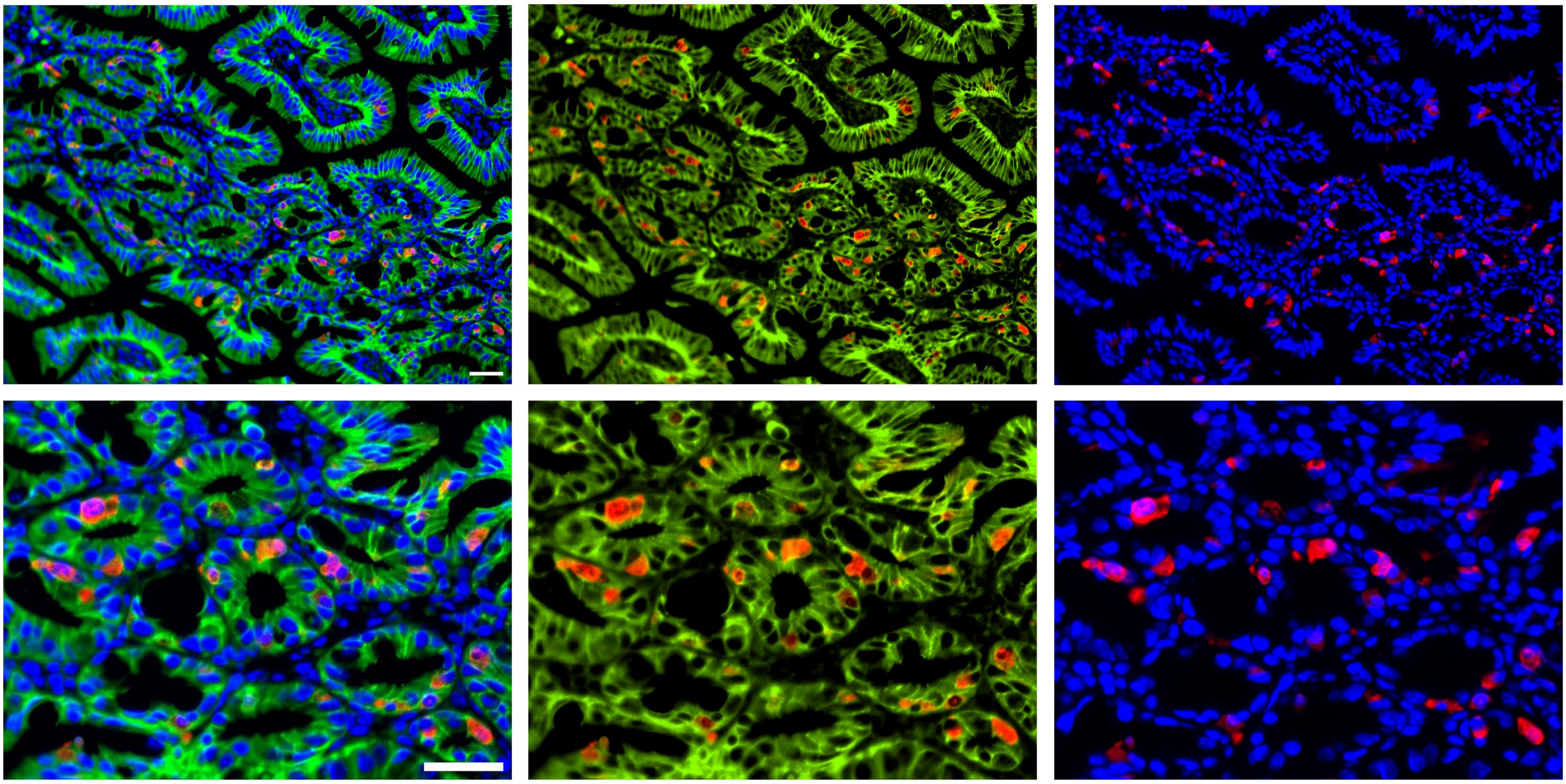

Supplemental Figure 6: **Expression of cleaved caspase 3 in intestinal epithelial cells from CML mice.** (**a,b**). Immunofluorescence staining showing E-cadherin (green), cleaved caspase 3 (red) and DAPI (blue) expression in intestinal epithelial cells in the colon (panel **a**) and ileum (Panel **b**) of CML mice. Magnification is 20X. Scale bar is 30 µM.

Supplemental Figure 7

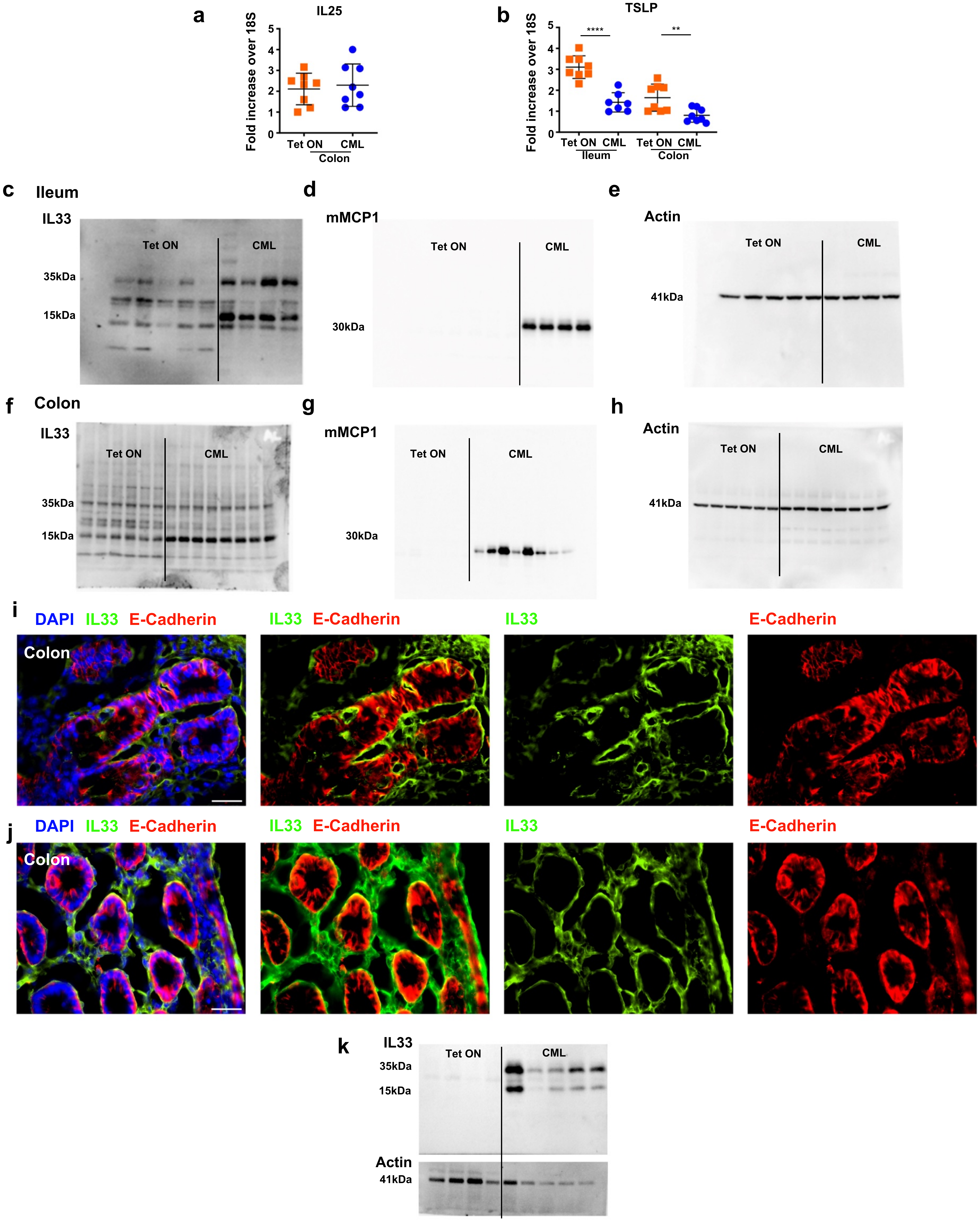

Supplemental Figure 7: **IL-33 is produced in the intestinal epithelium in CML mice.** (**a-k**). Lethally irradiated FVB mice were transplanted with SCLtTa/bcr-abl BM and maintained on or off Tet. Colon and ileum samples were harvested on day 21 (A-B) or 30 (C-K) post-transplantation. (**a,b**). mRNA gene expression of IL-25 in the colon (panel **a**), and TSLP in the ileum and colon (panel **b**) of CML animals. Data are from two experiments (n=8 mice/group). (**c-h**). Immunoblots of IL-33 (long and short forms), mMCP1 and actin in the ileum (panels **c-e**) and colon (panels **f-h**) of CML mice. (**i,j**) Immunofluorescence staining in the colon showing IL-33, E-cadherin, and merged expression of IL-33, E-cadherin and DAPI. Longitudinal (panel **i**) and cross sectional (panel **j**) tissue images are depicted. Scale bar is 30 μM. (**k**). Immunoblot of IL-33 (long and short forms) in intestinal epithelial cells in the colon of CML mice. Data are from two experiments (n=3-8 mice/group). Vertical lines on western blots denote noncontiguous gel lanes. Data are shown as mean ± SD. Pair wise statistical comparisons were performed using Welch's t test. $^{**}p<0.01$, $^{****}p<0.0001$.

Supplemental Figure 8

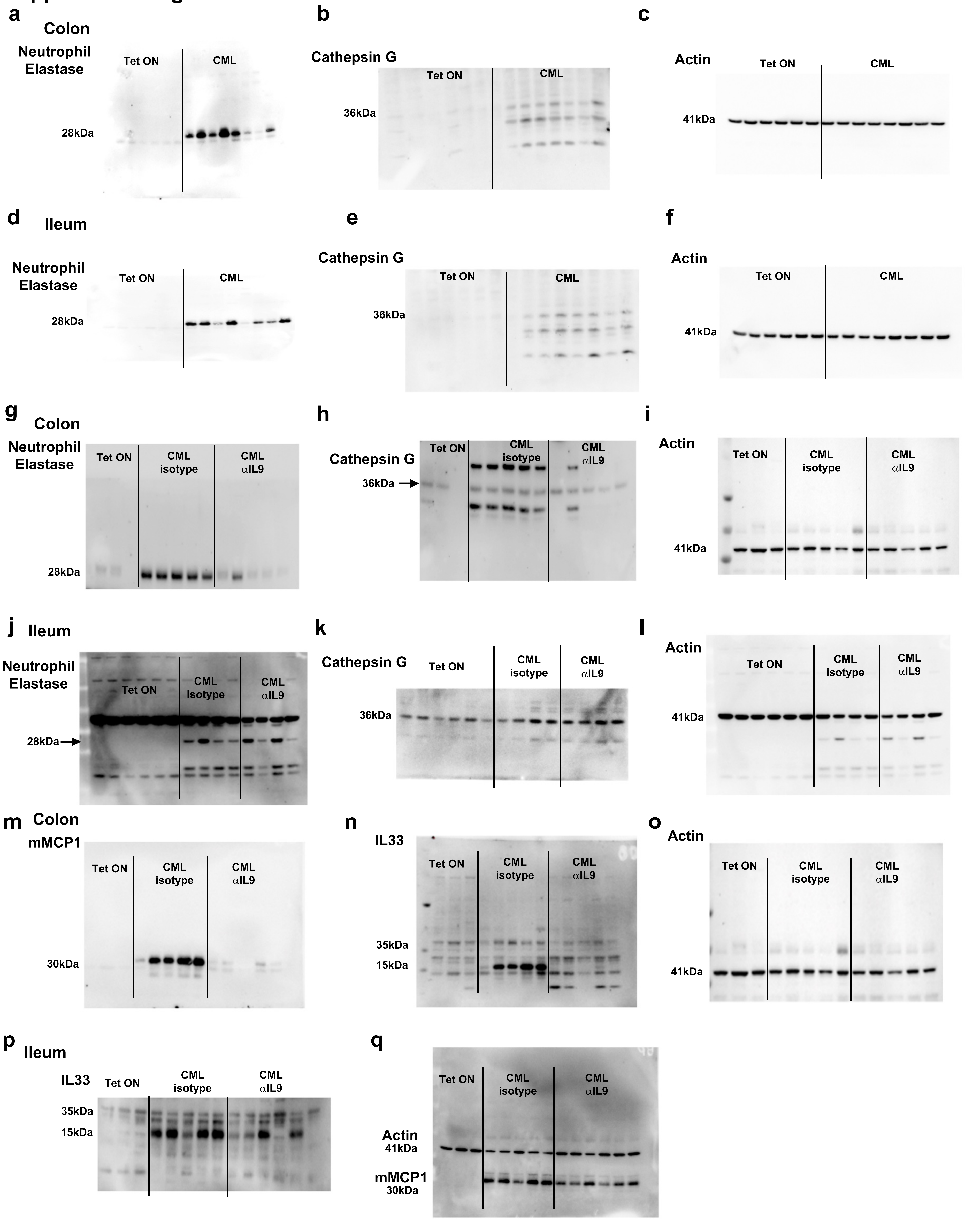

Supplemental Figure 8: **Blockade of IL-9 signaling preferentially reduces the production of chymase in the GI tract.** (**a-f**). Lethally irradiated FVB mice (n=3-8/group) were transplanted with SCLtTa/bcr-abl BM and maintained on or off Tet. Colon and ileum samples were harvested on day 30 post-transplantation. Immunoblots of neutrophil elastase, Cathepsin G and actin in the colon (panels **a-c**) and ileum (panels **d-f**) of CML mice. (**g-q**). Lethally irradiated FVB mice (n=3-5/group) were transplanted with SCLtTa/bcr-abl BM and maintained on or off Tet. Animals taken off Tet were treated with anti-IL-9 antibody or an isotype control three times per week for three weeks. Colon and ileum tissue extracts were collected 21 days post transplantation. Immunoblots of neutrophil elastase, Cathepsin G and actin in the colon (panels **g-i**) and ileum (panels **j-l**). Immunoblots of mMCP-1, IL-33 and actin in the colon (panels **m-o**) and IL-33, mMCP-1, and actin in the ileum (panels **p,q**). Vertical lines on western blots denote noncontiguous gel lanes.

Supplemental Figure 9

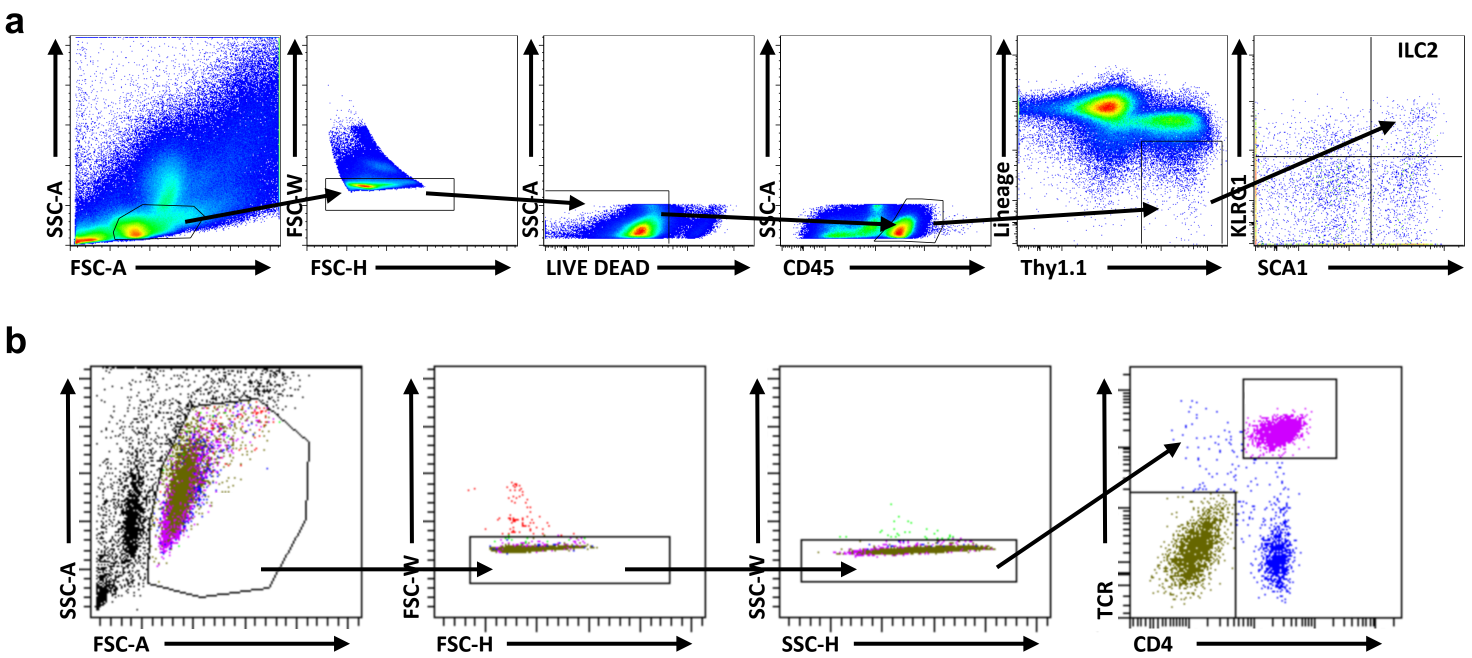

<u>Supplemental Figure 9</u>: **Gating strategies employed for cell sorting and flow cytometry data analysis.** (**a**). Gating strategy to detect type 2 innate lymphoid cells from the mesenteric lymph nodes and colon of lethally irradiated FVB mice were transplanted with BM cells from SCLtTa/bcr-abl animals and maintained on or off off Tet (CML) presented in Figure 6i. (**b**). Gating strategy to sort $CD4^{+}$ $TCR\beta^{+}$, $CD4^{+}$ $TCR\beta^{-}$ and $CD4^{-}$ $TCR\beta^{-}$ cells from the mesenteric lymph nodes of CML mice as depicted in Figure 6c.

SUPPLEMENTARY TABLE 1: FLOW CYTOMETRY ANTIBODIES AND OTHER REAGENTS

Target	Clone number	Fluorochrome	Catalog number	Dilution	Vendor
BrdU	N/A	biotin	ab2284	1:150	Abcam
CD11b	M1/70	e450	48-0112-82	1:50	eBioscience
CD11b	M1/70	APC	17-0112-82	1:50	eBioscience
CD11b	M1/70	APCCy7	5610393D	1:50	BD Pharmingen
CD11c	HL3	PE	557401	1:100	BD Pharmingen
CD4	RM4-5	e450	48-0042-82	1:25	eBioscience
CD45.1	A20	PE	553776	1:50	BD Pharmingen
CD64	X54-5/7.1	APC	17-0641-82	1:100	Invitrogen
Cleaved Caspase-3	5A1E	N/A	9664S	1:3000	Cell Signaling
Cathepsin G	N/A	N/A	PA5-89049	1:3000	Invitrogen
Gr-1	RB6-8C5	APC	553129	1:100	BD Pharmingen
Hematopoietic lineage cocktail	N/A	e450	88-7772-72	15ul/sample	Invitrogen
KLRG1	2F1	APCe780	47-5893-82	1:50	Invitrogen
Live/Dead fixable aqua	N/A	Pacific Orange	L34966	1ul/sample	Invitrogen
Ly6G	1A8	APCCy7	127623	1:100	Biolegend
Lysozyme	EPR2994(2)	N/A	ab108508	1:200	Abcam
Lysozyme	BGN/0696/5B1	N/A	MA182873	1:200	Invitrogen
Sca-1 (Ly-6A/E)	D7	PerCP-Cy5	45-5981-80	1:50	Invitrogen
TCRβ	H57-597	APC	553174	1:100	BD Pharmingen
TCRβ	H57-597	FITC	553171	1:100	BD Pharmingen
TCRβ	H57-597	BUV737	367-5961-82	1:100	eBioscience
Thy1.1	OX-7	FITC	554897	1:100	BD Pharmingen
Dnk anti-mouse	N/A	AF488	ab150109	1:300	Abcam
E-Cadherin	4A2	N/A	14472S	1:200	Cell Signaling
E-Cadherin	N/A	N/A	20874-1-A	1:200	Proteintech
Goat anti-mouse	N/A	AF594	ab150116	1:300	Abcam
Goat anti-rabbit	N/A	AF594	ab150080	1:300	Abcam
Goat anti-rabbit	N/A	AF488	ab6717	1:500	Abcam
Goat anti-rat	N/A	Cy5	ab6565	1:300	Abcam
Goat anti-rabbit	N/A	biotin	A16122	1:100	Thermo Scientific
I-A[b]	AF6-120.1	FITC	553551	1:50	BD Pharmingen
Mouse anti-rat	N/A	biotin	13-4813-85	1:100	Invitrogen
Mouse IgG1	MOPC-21	N/A	BE0083	N/A	BioXCell
Mouse IgG2a	C140SF9	N/A	N/A	N/A	N/A
Mouse IgG2a	C1.18.4	N/A	BE0085	N/A	BioXCell
MPTXs	EPR20920-19	N/A	ab238123	1:150	Abcam
Neutrophil Elastase	E8U3X	N/A	90120	1:3000	Cell Signaling
Rat IgG	N/A	N/A	012-000-003	N/A	Jackson Immuno Res
Purified anti-CD4	GK1.5	N/A	BE0003-1	N/A	BioXCell
Purified anti-IL5	TRFK5	N/A	BE0198	N/A	BioXCell
Purified anti-IL9	MM9C1	N/A	N/A	N/A	N/A
Purified anti- IL13		N/A	Bs-0560R	N/A	UCB Pharma
Purified anti-IL25	2C3.2	N/A	N/A	N/A	N/A
Purified anti-GATA3	L50-823	N/A	558686	1:200	BD Pharmingen
Purified anti-KLRG1	2F1	N/A	562190	1:150	BD Pharmingen
Purified anti-ST2	245707	N/A	MAB10041-500	N/A	R&D

Rabbit anti-mouse DCAMKL-1	N/A	N/A	ab31704	1:50	Abcam
Rabbit anti-mouse IL9	EPR23484-151	N/A	ab227027	1:200	Abcam
Rat anti-mouse MCP-1	RF6.1	N/A	14-5503-82	1:200	Invitrogen
Rat anti-mouse CD3	CD3-12	N/A	ab11089	1:200	Abcam
Rat anti-mouse IL33	396118	N/A	MAB3626	1:1000	R&D
Rat anti-mouse IL33	Polyclonal	N/A	AF3626	1:200	R&D
Rat anti-mouse MCP-1/Mcpt1	Polyclonal	N/A	MAB5146-SP	1:1000	R&D
Mouse anti-ß-actin	E4D9Z	N/A	4970S	1:5000	Cell Signaling

* APC=Allophycocyanin, AF=AlexaFluor, Cy=Cyanine, PE= Phycoerythrin, FITC= Fluorescein Isothiocyanate, PerCP= Peridinin-Chlorophyll-protein

SUPPLEMENTARY TABLE 2: RT-QPCR PRIMERS (LISTED 5'-3')

Target	Forward	Reverse
Areg	GCAGATACATCGAGAACCTGG	CTGCAATCTTGGATAGGTCCTTG
Crypt1	TCAAGAGGCTGCAAAGGAAGAGAAC	TGGTCTCCATGTTCAGCGACAGC
DCLK	CAAGCCAGCCATGTCGTTC	TTCCTTTGAAGTAGCGGTCAC
Fcer1a	GCCCCGTCTCCATTAG	CAATAACCCCGTGTCC
GMCSF	TTTACTTTTCCTGGGCAT	TAGCTGGCTGTCATGTTCAA
Gob-5	ACTAAAGGTGGCCTACCTCCAA	GGAGGTGACAGTCAAGGTGAG
IL13	GCTTATTGAGGAGCTGAGCAACA	GGCCAGGTCCACACTCCATA
IL17rb	CCATCCCTCCAGATGACAAC	TGCTCCTTCCTTGCCTCCAAGTTA
IL22	TCTGAGAAATGCTTGCGTCTGA	ACTGAGCCAGGTTTCATGTGAA
IL25	ACAGGGACTTGAATCGGGTC	TGGTAAAGTGGGACGGAGTTG
IL33	GGTGTGGATGGGAAGAAGCTG	GAGGACTTTTTGTGAAGGACG
IL4	ATCATCGGCATTTTGAACGAGGTC	ACCTTGGAAGCCCTACAGACG
IL5	GATGAGGCTTCCTGTCCCTACT	TGACAGGTTTTGGAATAGCATTTCC
IL6	GTTXCTCTGGGAAATCGTGGA	TCCAGTTTGGTAGCATCCATC
IL9	CATCAGTGTCTCTCCGTCCCAACTGATG	GATTTCTGTGTGGCATTGGTCAG
IL9R	ATGGGACAGGAACAGGTCAG	AGGTCACTCCAACGATACGG
IFNγ	TCAAGTGGCATAGATGTGGAAGAA	TGGCTCTGCAGGATTTTCATG
mMCPs (1,2,4chymase)	GCTGGAGCTGAGGAGATT	GGTGAAGACTGCAGGGG
mMCP-7 (Tryptase)	CCTCACTGTGTCCAAATGCTA	CCTCCTGCCTCAGAGACC
Sucnr1	GGGGACCTATGGAGATGTTCT	GCCAGCGAGATTAAAATGGCAA
sPLA2	AGGATTCCCCCAAGATGCCAC	CAGCCGTTTCTGACAGGAGTTCTGG
ST2	TCTCTTCTGGACCCTACCTCAG	TACTGCCCTCCGTAACTGTCA
TNFα	CTTCTGTCTACTGAACTTCGGG	CAGGCTTGTCACTCGAATTTTG
TSLP	AGCTTGTCTCCTGAAAATCGAG	AGGTTTGATTCAGGCAGATGTT
