# Supplementary material for: Interleukin-9 production by type 2 innate lymphoid cells induces Paneth cell metaplasia and small intestinal remodeling
Source: Nat Commun. 2023 Dec 2;14:7963. doi: 10.1038/s41467-023-43248-5 (PMC10693577; doi:10.1038/s41467-023-43248-5)
Supplement: Supplementary file 3 — Description of Additional Supplementary Files [file 41467_2023_43248_MOESM3_ESM.pdf]

Description of Additional Supplementary Files

File Name: Supplementary Data 1
Description: Fold change metabolites in the ileum of CML mice
