# Supplementary material for: Interleukin-9 production by type 2 innate lymphoid cells induces Paneth cell metaplasia and small intestinal remodeling
Source: Nat Commun. 2023 Dec 2;14:7963. doi: 10.1038/s41467-023-43248-5 (PMC10693577; doi:10.1038/s41467-023-43248-5)
Supplement: Supplementary file 5 — Reporting Summary [file 41467_2023_43248_MOESM5_ESM.pdf]

nature portfolio

Corresponding author(s): William Drobyski

Last updated by author(s): Oct 16, 2023

Reporting Summary

Nature Portfolio wishes to improve the reproducibility of the work that we publish. This form provides structure for consistency and transparency in reporting. For further information on Nature Portfolio policies, see our Editorial Policies and the Editorial Policy Checklist.

Statistics

For all statistical analyses, confirm that the following items are present in the figure legend, table legend, main text, or Methods section.

n/a	Confirmed	
☐	☒	The exact sample size (*n*) for each experimental group/condition, given as a discrete number and unit of measurement
☐	☒	A statement on whether measurements were taken from distinct samples or whether the same sample was measured repeatedly
☐	☒	The statistical test(s) used AND whether they are one- or two-sided<br>*Only common tests should be described solely by name; describe more complex techniques in the Methods section.*
☒	☐	A description of all covariates tested
☐	☒	A description of any assumptions or corrections, such as tests of normality and adjustment for multiple comparisons
☐	☒	A full description of the statistical parameters including central tendency (e.g. means) or other basic estimates (e.g. regression coefficient) AND variation (e.g. standard deviation) or associated estimates of uncertainty (e.g. confidence intervals)
☐	☒	For null hypothesis testing, the test statistic (e.g. *F*, *t*, *r*) with confidence intervals, effect sizes, degrees of freedom and *P* value noted<br>*Give P values as exact values whenever suitable.*
☒	☐	For Bayesian analysis, information on the choice of priors and Markov chain Monte Carlo settings
☐	☒	For hierarchical and complex designs, identification of the appropriate level for tests and full reporting of outcomes
☒	☐	Estimates of effect sizes (e.g. Cohen's *d*, Pearson's *r*), indicating how they were calculated

Our web collection on statistics for biologists contains articles on many of the points above.

Software and code

Policy information about availability of computer code

Data collection	All flow cytometry data were acquired on an LSR-II or Fortessa X-20 cytometer.<br>Metabolon software on a web-service platform utilizing Microsoft,NET technologies was used to collect data for metabolite analysis.
Data analysis	Flow cytometry data was analyzed using FlowJo (Version 9).<br>Metabolon performed data analysis for metabolite experiments. Metabolon data analysts used proprietary visualization and interpretation software to confirm the consistency of peak identification among the various samples.

For manuscripts utilizing custom algorithms or software that are central to the research but not yet described in published literature, software must be made available to editors and reviewers. We strongly encourage code deposition in a community repository (e.g. GitHub). See the Nature Portfolio guidelines for submitting code & software for further information.

Data

Policy information about availability of data

All manuscripts must include a data availability statement. This statement should provide the following information, where applicable:

- Accession codes, unique identifiers, or web links for publicly available datasets
- A description of any restrictions on data availability
- For clinical datasets or third party data, please ensure that the statement adheres to our policy

The authors declare that all data pertaining to the current study are available within the article and Supplementary Information or available from the corresponding author upon request. Source data are provided with this paper.

Research involving human participants, their data, or biological material

Policy information about studies with human participants or human data. See also policy information about sex, gender (identity/presentation), and sexual orientation and race, ethnicity and racism.

Reporting on sex and gender	*Use the terms sex (biological attribute) and gender (shaped by social and cultural circumstances) carefully in order to avoid confusing both terms. Indicate if findings apply to only one sex or gender; describe whether sex and gender were considered in study design; whether sex and/or gender was determined based on self-reporting or assigned and methods used. Provide in the source data disaggregated sex and gender data, where this information has been collected, and if consent has been obtained for sharing of individual-level data; provide overall numbers in this Reporting Summary. Please state if this information has not been collected. Report sex- and gender-based analyses where performed, justify reasons for lack of sex- and gender-based analysis.*
Reporting on race, ethnicity, or other socially relevant groupings	*Please specify the socially constructed or socially relevant categorization variable(s) used in your manuscript and explain why they were used. Please note that such variables should not be used as proxies for other socially constructed/relevant variables (for example, race or ethnicity should not be used as a proxy for socioeconomic status). Provide clear definitions of the relevant terms used, how they were provided (by the participants/respondents, the researchers, or third parties), and the method(s) used to classify people into the different categories (e.g. self-report, census or administrative data, social media data, etc.) Please provide details about how you controlled for confounding variables in your analyses.*
Population characteristics	*Describe the covariate-relevant population characteristics of the human research participants (e.g. age, genotypic information, past and current diagnosis and treatment categories). If you filled out the behavioural & social sciences study design questions and have nothing to add here, write "See above."*
Recruitment	*Describe how participants were recruited. Outline any potential self-selection bias or other biases that may be present and how these are likely to impact results.*
Ethics oversight	*Identify the organization(s) that approved the study protocol.*

Note that full information on the approval of the study protocol must also be provided in the manuscript.

Field-specific reporting

Please select the one below that is the best fit for your research. If you are not sure, read the appropriate sections before making your selection.

☒ Life sciences ☐ Behavioural & social sciences ☐ Ecological, evolutionary & environmental sciences

For a reference copy of the document with all sections, see nature.com/documents/nr-reporting-summary-flat.pdf

Life sciences study design

All studies must disclose on these points even when the disclosure is negative.

Sample size	No statistical models were used to predetermine sample size. Nearly all of the experimental interventional group comparisons had 7-15 mice per group which has typically been sufficient to detect statistically significant differences between groups. All but a few of the experimental endpoints that were assessed and are presented in over 100 panels in the manuscript and supplemental data were repeated at least twice.
Data exclusions	No data were excluded from the manuscript.
Replication	All experiments were replicated in at least two individual independent experiments.
Randomization	All groups were age and sex matched.
Blinding	Blinding was not applicable. Mice with CML lose weight, have decreased activity and ruffled fur which distinguishes them from non-leukemic control animals so that blinding is not possible.

Reporting for specific materials, systems and methods

We require information from authors about some types of materials, experimental systems and methods used in many studies. Here, indicate whether each material, system or method listed is relevant to your study. If you are not sure if a list item applies to your research, read the appropriate section before selecting a response.

Materials & experimental systems

n/a	Involved in the study
☐	☒ Antibodies
☒	☐ Eukaryotic cell lines
☒	☐ Palaeontology and archaeology
☐	☒ Animals and other organisms
☒	☐ Clinical data
☒	☐ Dual use research of concern
☒	☐ Plants

Methods

n/a	Involved in the study
☒	☐ ChIP-seq
☐	☒ Flow cytometry
☒	☐ MRI-based neuroimaging

Antibodies

Antibodies used

Target Clone number Fluorochrome Catalog number Dilution Vendor
BrdU N/A biotin ab2284 N/A Abcam
CD11b M1/70 e450 48-0112-82 1:20 eBioscience
CD11b M1/70 APC 17-0112-82 1:20 eBioscience
CD11b M1/70 APCCy7 5610393D 1:20 BD Pharmingen
CD11c HL3 PE 557401 1:50 BD Pharmingen
CD4 RM4-5 e450 48-0042-82 1:50 eBioscience
CD45.1 A20 PE 553776 1:50 BD Pharmingen
CD64 X54-5/7.1 APC 17-0641-82 1:50 Invitrogen
Cleaved Caspase-3 5A1E N/A 9664S 1:3000 Cell Signaling
Cathepsin G N/A N/A PA5-89049 1:3000 Invitrogen
Gr-1 RB6-8C5 APC 553129 1:50 BD Pharmingen
Hematopoietic lineage cocktail N/A e450 88-7772-72 1:50 Invitrogen
KLRG1 2F1 APCe780 47-5893-82 1:50 Invitrogen
Live/Dead fixable aqua N/A Pacific Orange L34966 N/A Invitrogen
Ly6G 1A8 APCCy7 127623 1:50 Biolegend
Lysozyme EPR2994(2) N/A ab108508 1:200 Abcam
Lysozyme BGN/0696/5B1 N/A MA182873 1:200 Invitrogen
Sca-1 (Ly-6A/E) D7 PerCP-Cy5 45-5981-80 1:50 Invitrogen
TCRβ H57-597 APC 553174 1:50 BD Pharmingen
TCRβ H57-597 FITC 553171 1:50 BD Pharmingen
TCRβ H57-597 BUV737 367-5961-82 1:50 eBioscience
Thy1.1 OX-7 FITC 554897 1:50 BD Pharmingen
Dnk anti-mouse N/A AF488 ab150109 1:300 Abcam
E-Cadherin 4A2 N/A 14472S 1:200 Cell Signaling
E-Cadherin N/A N/A 20874-1-A 1:200 Proteintech
Goat anti-mouse N/A AF594 ab150116 1:300 Abcam
Goat anti-rabbit N/A AF594 ab150080 1:300 Abcam
Goat anti-rabbit N/A AF488 Ab150077 1:300 Abcam
Goat anti-rabbit N/A FITC ab6717 1:300 Abcam
Goat anti-rat N/A Cy5 ab6565 1:300 Abcam
Goat anti-rabbit N/A biotin 1:300 Thermo Scientific
I-A[b] AF6-120.1 FITC 553551 1:50 BD Pharmingen
Mouse anti-rat N/A biotin 13-4813-85 1:300 Invitrogen
Mouse IgG1 MOPC-21 N/A BE0083 N/A BioXCell
Mouse IgG2a C140SF9 N/A N/A N/A N/A
Mouse IgG2a C1.18.4 N/A BE0085 N/A BioXCell
MPTXs EPR20920-19 N/A ab238123 1:200 Abcam
Neutrophil Elastase E8U3X N/A 90120 1:3000 Cell Signaling
Rat IgG N/A N/A 012-000-003 N/A Jackson Immuno Res
Purified anti-CD4 GK1.5 N/A BE0003-1 N/A BioXCell
Purified anti-IL5 TRFK5 N/A BE0198 N/A BioXCell
Purified anti-IL9 MM9C1 N/A N/A N/A N/A
Purified anti- IL13 N/A Bs-0560R N/A UCB Pharma
Purified anti-IL25 2C3.2 N/A N/A N/A N/A
Purified anti-GATA3 L50-823 N/A 558686 1:200 BD Pharmingen
Purified anti-KLRG1 2F1 N/A 562190 1:200 BD Pharmingen
Purified anti-ST2 245707 N/A MAB10041-500 N/A R&D
Rabbit anti-mouse DCAMKL-1 N/A N/A ab31704 1:200 Abcam
Rabbit anti-mouse IL9 EPR23484-151 N/A ab227027 1:200 Abcam

Rat anti-mouse MCP-1 RF6.1 N/A 14-5503-82 1:200 Invitrogen
Rat anti-mouse CD3 CD3-12 N/A ab11089 1:200 Abcam
Rat anti-mouse IL33 396118 N/A MAB3626 1:1000 R&D
Rat anti-mouse MCP-1/Mcpt1 Polyclonal N/A MAB5146-SP 1:1000 R&D
Mouse anti-ß-actin E4D9Z N/A 4970S 1:5000 Cell Signaling

Validation

All antibodies were validated by the suppliers based on manufacturers descriptions and data sheets. In house antibodies were validated by other peer-reviewed publications.

The following references validate the use of in house antibodies and are cited in the manuscript: Ballantyne SJ, Barlow JL, Jolin HE, et al. Blocking IL-25 prevents airway hyperresponsiveness in allergic asthma. J. Allergy Clin. Immunol. 120, 1324-1331 (2007); Berry LM, Adams R, Airey M, et al. In vitro and in vivo characterization of anti-murine IL-13 antibodies recognizing distinct functional epitopes. Int. Immunopharm. 9, 201-206 (2009); and Wilhelm C, Hirota K, Stieglitz B, et al. An IL-9 fate reporter demonstrates the induction of an innate IL-9 response in lung inflammation. Nat. Immunol. 12, 1071-1077 (2011).

Animals and other research organisms

Policy information about studies involving animals; ARRIVE guidelines recommended for reporting animal research, and Sex and Gender in Research

Laboratory animals

FVB (H-2q) mice were bred in the Animal Resource Center at the Medical College of Wisconsin (MCW) or purchased from Jackson Laboratories (Bar Harbor, ME) (Stock number #001800). Transgenic mice in which a tetracycline-controlled transactivator was placed under the control of the murine stem cell leukemia gene 3' enhancer [SCLtTA mice (FVB background)] were crossbred to transgenic TRE-BCR-ABL mice (FVB background) which expressed the bcr/abl oncogene to create double transgenic SCLtTA x BCR-ABL mice (i.e., CML mice). Mice were 6-10 weeks of age when used in experiments. Animals were housed under specific pathogen free conditions.

Wild animals

No wild animals were used.

Reporting on sex

Both male and female mice were used. Donor and recipient mice were sex matched in all experiments.

Field-collected samples

No field collected samples were used.

Ethics oversight

Ethics oversight was performed by the Institutional Animal Care and Use Committee (IACUC) at the Medical College of Wisconsin.

Note that full information on the approval of the study protocol must also be provided in the manuscript.

Flow Cytometry

Plots

Confirm that:

☒ The axis labels state the marker and fluorochrome used (e.g. CD4-FITC).

☒ The axis scales are clearly visible. Include numbers along axes only for bottom left plot of group (a 'group' is an analysis of identical markers).

☒ All plots are contour plots with outliers or pseudocolor plots.

☒ A numerical value for number of cells or percentage (with statistics) is provided.

Methodology

Sample preparation

Splenocytes were obtained by grinding the tissues though a mesh screen with a syringe plunger. Red blood cells in the cell suspension were lysed with Ammonium Chloride Tris (ACT) lysis buffer, prepared with ammonium chloride solution and Tris-HCl solution, pH 7.2. The cell suspension was subsequently filtered through a cell strainer and prepared for further analysis.

Epithelial cells were isolated from colon samples in the pre-digestion buffer using the Lamina Propria Dissociation Kit (Miltenyi Biotec, Auburn, CA) according to the manufacturer's instructions. To isolate lymphocytes from the lamina propria, colon samples were first washed in DMEM medium with DTT and EDTA. Samples were then digested with 10µg/ml liberase TL (Roche, Basel, Switzerland) and 0.05% DNase (QIAGEN, Hilden, Germany), and processed using the gentleMACS Dissociator (Miltenyi). The resulting cell suspension was then layered on a 44%/67% Percoll gradient (Sigma).

Instrument

Flow cytometry data were acquired on an LSRII (BD Biosciences, CA)

Software

All flow cytometry data were acquired using BD FacsDiva.

Cell population abundance

For FACS sorting experiments, post sorting purity was performed and was greater than 90%.

Gating strategy	A preliminary FSC/SSC gate was utilized to gate on the morphology of lymphocytes. Then, a single cell gate (FSC-A vs FSC-H) was used to exclude doublets. From this population, relevant gating strategies for each cell type are described in Supplemental Figure 9 in the paper.

☒ Tick this box to confirm that a figure exemplifying the gating strategy is provided in the Supplementary Information.
